# Simulated Microgravity-Induced Changes to Drug Response in Cancer Cells Quantified Using Fluorescence Morphometry

**DOI:** 10.3390/life13081683

**Published:** 2023-08-04

**Authors:** Spencer McKinley, Adam Taylor, Conner Peeples, Megha Jacob, Gargee Khaparde, Yohan Walter, Andrew Ekpenyong

**Affiliations:** 1 Biology Department, Creighton University, Omaha, NE 68178, USA; spencermckinley@creighton.edu (S.M.); adamtaylor@creighton.edu (A.T.); meghajacob@creighton.edu (M.J.); gargeekhaparde@creighton.edu (G.K.); 2 Physics Department, Creighton University, Omaha, NE 68178, USA; connerpeeples@creighton.edu (C.P.); yohanwalter@creighton.edu (Y.W.)

**Keywords:** microgravity, chemotherapy, paclitaxel, hydroxyurea, morphometry, drug response, immune dysfunction, space medicine, terrestrial medicine, fluorescence microscopy

## Abstract

Unlike plants that have special gravity-sensing cells, such special cells in animals are yet to be discovered. However, microgravity, the condition of apparent weightlessness, causes bone, muscular and immune system dysfunctions in astronauts following spaceflights. Decades of investigations show correlations between these organ and system-level dysfunctions with changes induced at the cellular level both by simulated microgravity as well as microgravity conditions in outer space. Changes in single bone, muscle and immune cells include morphological abnormalities, altered gene expression, protein expression, metabolic pathways and signaling pathways. These suggest that human cells mount some response to microgravity. However, the implications of such adjustments on many cellular functions and responses are not clear. Here, we addressed the question whether microgravity induces alterations to drug response in cancer cells. We used both adherent cancer cells (T98G) and cancer cells in suspension (K562) to confirm the known effects of simulated microgravity and then treated the K562 cells with common cancer drugs (hydroxyurea and paclitaxel) following 48 h of exposure to simulated microgravity via a NASA-developed rotary cell culture system. Through fluorescence-guided morphometry, we found that microgravity abolished a significant reduction (*p* < 0.01) in the nuclear-to-cytoplasm ratio of cancer cells treated with hydroxyurea. Our results call for more studies on the impact of microgravity on cellular drug response, in light of the growing need for space medicine, as space exploration grows.

## 1. Introduction

As space exploration grows and with commercial space flights having begun, understanding the impact of microgravity on human physiology has become a more urgent question. Space medicine has always been fundamental to human exploration of space [1,2]. It is now expanding beyond its usual specialty within aerospace medicine, beyond being a discipline that tackles comprehensive health care delivery issues within the challenging environment of space into a discipline that uses that challenging environment for drug discovery and development for both terrestrial and space health applications [3,4,5]. The quest for personalized medicine for terrestrial purposes also becomes a quest for personalized medicine for space explorers [6,7]. Likewise, the current quest for quantifying drug response at the cellular level to expedite terrestrial personalized medicine [8] also merges with recent efforts to investigate the impact of microgravity on drug response at the cellular level [9]. Interestingly, a comprehensive literature review of the impact of microgravity on cancer cells also highlights the increasing attempts to use simulated microgravity as a tool for drug discovery and development [9]. Within the broader question of the impact of microgravity on cells is the more focused question: how do cells, cancer cells in particular, respond to drugs when subjected to microgravity? A comprehensive review of the literature in the last decade showed that little is known regarding how microgravity affects cellular and molecular events that determine physiological and biological responses in cancer cells [10]. A recapitulation of more recent literature in this regard is appropriate.

It has been shown that microgravity alters the effects of chemotherapeutic drugs on cancer cell migration [11,12]. The alterations were drug-dependent: leukemic cancer cells treated with daunorubicin showed increased chemotactic migration (*p* < 0.01) following simulated microgravity (µg) compared to normal gravity on earth (1 G). But the same cells treated with doxorubicin showed enhanced migration both in 1 G and following µg [11,12]. A different group of researchers replicated the major conclusion (namely, alteration of drug response by microgravity) from these findings [11,12] using doxorubicin and gastric cancer cells [13]. Remarkably, Rembiałkowska et al. found that microgravity increased the chemotherapeutic effect in the case of drug-resistant gastric cancer cells [13]. In particular, they found that following simulated microgravity, gastric cancer cells showed decreased expression of genes related to drug resistance and increased DNA/RNA damage marker expression. Moreover, they reported significant reorganization of F-actin fibers in these cells after exposure to microgravity conditions [13], and such morphological reorganization might be implicated in the reported altered (here, increased) sensitivity to chemotherapy. Similar cytoskeletal and morphological reorganization induced by microgravity was found to correlate with the reduction of cisplatin resistance in ovarian cancer cells [14].

Unsurprisingly, since all these reports [11,12,13,14] comparing the response of cancer cells to drugs in normal gravity on earth (1 G) versus microgravity conditions are very recent, there is a paucity of such reports in the literature. However, recent literature is replete with findings confirming previously reported changes in single cells, including cancer cells, due to microgravity conditions. Our major premise in this work is that the recently confirmed microgravity-induced changes in single cells may also lead to modulation of drug response in those cells. A quick survey of recently confirmed microgravity-induced alterations in cancer cells and cancer-related cells follows, to place our work within a broader context in space biomedicine.

Macrophages and other immune cells have emerged as major tools and targets in cancer therapy [15,16]. Macrophages, long known to be altered by microgravity [17], have been found to respond to microgravity in the International Space Station (ISS) in a matter of seconds [18]. Several reports showed that simulated microgravity alters the growth and differentiation of stem cells and cancer stem cells [19,20,21,22,23]. Recently, human blood-derived stem cells cultured under microgravity conditions in the ISS showed alterations in osteoblastic differentiation [24], in line with previous results from simulated microgravity. Grimm et al. provide a review of the literature on the effects of both simulated and real microgravity on the differentiation and growth of stem cells and cancer stem cells [25]. Cultured aboard the ISS for 5.5 weeks, human induced pluripotent stem cell-derived cardiomyocytes have confirmed microgravity-induced alterations to calcium handling [26] in cells. These newly confirmed alterations induced by microgravity in cancer and cancer-related cells have several intersections with currently known hallmarks of cancer [27,28,29,30] such as sustaining proliferative signaling, resisting cell death, enabling replicative immortality and activating invasion and metastasis. Since anti-cancer drugs use these hallmarks as therapeutic targets, direct or indirect alterations of these hallmarks by microgravity could engender changes in the response of cancer cells to these drugs.

To address the question whether confirmed changes that microgravity produces in cancer cells might cause alterations in the response of such cells to common cancer drugs, we selected two well characterized cancer cell lines: an adherent cell line, T98G [31,32], and cancer cells that grow in suspension, K562 [33,34], to confirm the known effects of microgravity via a NASA-developed and widely used rotary cell culture system (RCCS) [21,35,36,37]. The RCCS (Synthecon, Houston, TX, USA) is a 3D system that uses rotating high-aspect ratio vessels (HARV) to maintain cell cultures in low shear, continuous free fall and randomizing gravity vector [35,36,37,38]. Following 48 h of exposure to microgravity, we then treated the leukemic cells with two drugs used in the treatment of leukemia and other cancers: (1) hydroxyurea [39], which functions by inhibiting mitosis and thereby blocks cell growth, and (2) paclitaxel, which causes mitotic cell cycle arrest by stabilizing microtubules [40,41]. Using fluorescence-guided morphometry to visualize and quantify the microgravity-induced alterations and implications on drug response, we found an intriguing loss in the significant reduction (*p* < 0.01) in the nuclear-to-cytoplasm ratio of cancer cells treated with hydroxyurea. Changes found with paclitaxel were not statistically significant. Our results accentuate the need for further studies on the impact of microgravity on cancer cell drug responses in particular and cellular drug response in general, to prime space medicine for space exploration while advancing terrestrial medicine.

## 2. Materials and Methods

### 2.1. Cell Culture

We used the glioblastoma multiforme cell line isolated from a 61-year-old patient, T98G (ATCC^®^ CRL-1690™) and the lymphoblastic cell line isolated from the bone marrow of a 53-year-old chronic myelogenous leukemia patient and K562 (ATCC CCL-243) both purchased from the American Type Cell Culture Collection (ATCC, Manassas, VA, USA). T98G cells are adherent cells with a fibroblast morphology. The K562 cells grow in suspension. We followed specific protocols from ATCC in culturing both cell types, as reported in our previous works [12,40,42,43]. Briefly, we cultured K562 cells using RPMI 1640 (11875093, Life Technologies, Waltham, MA, USA), supplemented with 10% (*v*/*v*) fetal bovine serum (FBS) and 1% penicillin/streptomycin as growth medium. We cultured the T98G cells in Eagle’s Minimum Essential Medium (EMEM, Sigma Aldrich, St Louis, MI, USA, ATCC 30-2003) or Dulbecco’s Modified Eagle Medium (DMEM, Sigma Aldrich, St Louis, MI, USA, Corning 10-013-CMR) supplemented with 10% fetal bovine serum (FBS, Gibco, New York, NY, USA, 10100147) and 1% penicillin–streptomycin (P/S, Sigma Aldrich, St Louis, MI, USA, P4333-100ML). Both cell lines were maintained in an incubator kept at 95% air, 5% CO_2_ and a temperature of 37 °C. All experiments were performed when cells were in the logarithmic growth phase, usually at a viability of over 95%. Routine viability tests were done using trypan blue (Sigma-Aldrich, St Louis, MI, USA), manual counting with a hemocytometer or Invitrogen Countess II automatic cell counter (Thermo Fisher Scientific, Waltham, MA, USA, AMQAX1000).

### 2.2. Rotary Cell Culture System

**Figure 1:**
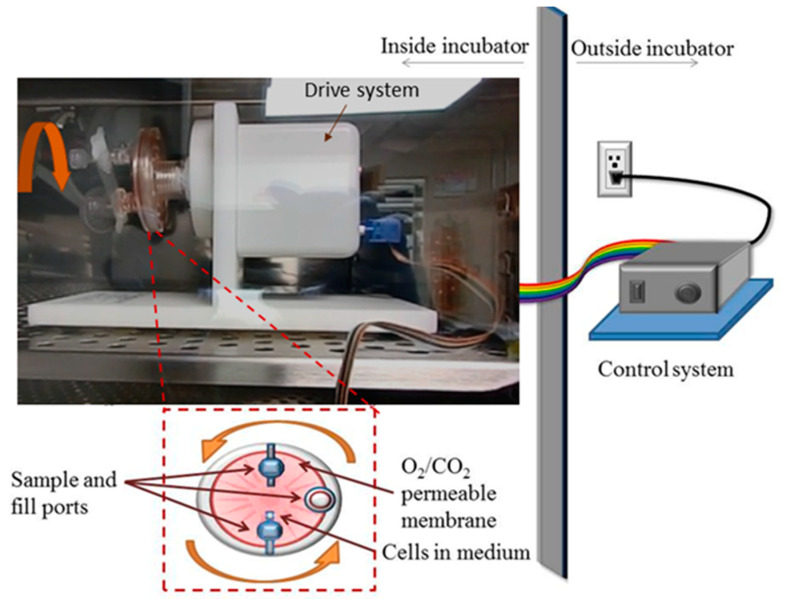
Schematic and picture of the rotary cell culture system, RCCS, used in the simulation of microgravity inside a cell culture incubator. The picture shows the drive system along with a side view of the cell culture vessel. The schematic presents a front view of the 10 mL cell culture vessel that is rotated to produce a time-averaged microgravity condition.

Simulated microgravity conditions were produced using the rotary cell culture system, RCCS™ (Figure 1). RCCS was developed at the Johnson Space Center by NASA. We purchased a commercially available version from Synthecon^®^ Inc. (Houston, TX, USA). The RCCS not only simulates microgravity but also serves as a 3D cell culture technology for culturing both suspension and anchorage-dependent cells [13,35,37]. The RCCS is a bioreactor equipped with a 10 mL or 50 mL disposable high aspect ratio vessel (HARV), with a silicon membrane on one side that provides gas exchange. It is designed to provide a low-shear cell culture system. Via vertical rotation around a horizontal axis, the RCCS produces rigid-body rotation of the entire HARV and the cell culture medium, thereby randomizing the gravity vector to simulate microgravity [12,22,37]. Ideally, a rotational control could be added where the HARV rotates horizontally around a vertical axis as a further check for the effect of rotation-induced shear stress, as done by Mylabathula et al. [38]. Thus, our current experimental design does not fully decouple the possible effects of rotation-induced shear stress from the effects of microgravity. We seeded T98G and K562 cells at a concentration of 2 × 10^5^ cells/mL in T-25 flasks (as static controls at normal gravity, 1 G) and in 10 mL HARVs and rotated at 15 rpm as microgravity condition, µg. This rotation speed is the reported optimal speed for many cell types [13,36,37] including K562 and T98G cells, in accordance with the manufacturer’s protocols. Cells were maintained in this microgravity condition for 48 h (K562) or 72 h (T98G) inside the same incubator (Figure 1) as the normal gravity or 1 G cells. The T98G cells were used for functional verification of our simulated microgravity conditions, while the K562 cells were used to assess drug response. Although 3D spheroids of T98G cells were formed in 48 h, the 72 h condition produced more and larger spheroids. The 48 h of microgravity for the K562 cells initially seeded at 2 × 10^5^ cells/mL was chosen for the introduction of drugs so as to coincide with the logarithmic growth phase of K562 cell density and viability, in light of its doubling time. At 48 h (both 1 G and µg), the cell density was about 1 × 10^6^ cells/mL and viability was consistently over 96% (see Table S1), both optimal for the pharmacological experiments.

### 2.3. Pharmacological Interventions

Following 48 h of exposure to microgravity µg, the K562 leukemic cells were treated with two chemotherapeutic drugs used against leukemia and other cancers, namely, hydroxyurea and paclitaxel. Hydroxyurea is an antitumor and antileukemic agent that functions by inhibiting mitosis and thereby blocking cell growth [39]. Paclitaxel also functions by blocking cell growth through mitotic cell cycle arrest achieved by stabilizing microtubules [40,41]. The final concentration of paclitaxel (Sigma-Aldrich 580555) was 5 μM. The final concentration of hydroxyurea (Sigma-Aldrich, H8267) was 100 μM. Cells were incubated for 24 h following drug treatments, before fluorescence microscopy was carried out, to assist with morphometry.

### 2.4. Fluorescence Microscopy

Fluorescence microscopy of cells was carried out using a Zeiss Vert.A1 AXIO fluorescence microscope with an inserted USB microscope camera (AmScope MU300), captured using the AmScope (86×) image capture program. The fluorescent dyes Hoechst 33342 (ThermoFisher Scientific) and Calcein AM (Invitrogen by ThermoFisher Scientific) were used to stain the cells for imaging. Hoechst 33342 is a blue (460–490 nm emission) fluorescent dye used for staining cellular DNA [44,45]. Calcein AM is an initially nonfluorescent dye that is membrane-permeable, but upon uptake into the cell, is degraded partially, making the dye membrane-impermeable. The degradation also results in the conversion from a nonfluorescent solution to green fluorescence (515 nm peak emission). The dye is thus retained selectively in cells with intact membranes and is often used as a measure of cell viability [46], as it stains the whole cytoplasm. Hoechst 3342 was used at a final concentration of 0.1 mM, while Calcein was used at a concentration of 1 µg/mL. Various treatment conditions of both T98G and K562 cells were stained, incubated and imaged as the control and also following other experimental conditions such as 48 h post-microgravity for K562 cells and 78 h post-microgravity for T98G. The T98G cells were imaged both in the adherent state (1 G) and in the suspended state following microgravity-induced formation of tissue spheroids.

Prior to imaging, the dyes were added directly to the culture medium at the concentrations noted previously. Each dye was incubated with the cells for 5–10 min. Once absorbed, the cells were washed twice with 2.0 mL of PBS to rinse away excess dye for optimal visual contrast. Cells were imaged while in 1.0 mL of PBS to prevent drying. The Hoechst 33342 DNA stain was imaged under a blue filter, while Calcein was imaged under a green filter to selectively view their emissions.

### 2.5. Fluorescence-Guided Morphometry

Fluorescence images of cells were analyzed qualitatively and quantitatively using freely available ImageJ software, version 1.53m (National Institute of Heath, NIH) and user-developed plugins. ImageJ is widely used in a broad range of applications requiring image analysis. Built-in techniques and plugins have steadily optimized acquisition of image-based measurements, from simple parameters such as length and area, to more complex analyses, some catered to highly specific applications. For our purposes, image analysis was used to gather morphological data and to detect changes in cell characteristics following treatment. Morphological changes are among well-established readouts of cytotoxicity/drug responses [47,48]. Though these effects could be visualized qualitatively, quantitative analysis was carried out using the following parameters:

1. Lacunarity, Λ.

Lacunarity, also referred to as texture, inhomogeneity or “gappiness,” is a measure of heterogeneity in images. As heterogeneity or the number and size of gaps in a region of interest increase, so does the lacunarity. This quantity has been used in numerous studies, including our previous work [40] as an indicator of granularity or inhomogeneity in cell morphology potentially caused by drug treatments. In this work, lacunarity was used to evaluate the heterogeneity in cell cytoplasm and nuclei following various treatments. Cells were first dyed with Hoechst 33342 to enhance the morphometry. Lacunarity was measured using ImageJ’s FracLac plugin. Cells imaged under 40× magnification were used in the analyses.

In its most basic form, lacunarity is calculated as:

(1) $$\lambda_{\varepsilon ,g}={\left(\frac{\sigma_{\varepsilon ,g}}{\mu_{\varepsilon ,g}}\right)}^{2},$$

where $\lambda_{\varepsilon ,g}$ is the lacunarity, ε is the caliber or size of the “box” in a grid for counting pixels via the “box counting” method, *g* is the position in a grid overlaying the image and the lacunarity was calculated by dividing the standard deviation $\sigma_{\varepsilon ,g}$ of the pixel values per box by the mean $\mu_{\varepsilon ,g}$ and squaring the quotient. The lacunarity was thus based on variations in pixel value for set box sizes throughout the image. In addition to presenting the measured values for lacunarity, FracLac has a built-in function that shows the dependence of the lacunarity on the box size. For our work, we used lacunarity to measure the complexity of images of cells, where changes in complexity may have been the direct result of treatment.

2. Circularity.

Circularity, *C*, is a shape descriptor defined as:

(2) $$C=\frac{4\pi A}{P^{2}},$$

where *A* is the area and *P* is the perimeter of the object (cell). The circularity ranges from 0 for an infinitely elongated polygon to 1 for a perfect circle. Due to increased computational power and ease of automatic calculation, circularity is now increasingly used for cell morphometry in diverse biomedical contexts from high-throughput cell sorting for clinical diagnostics using flow cytometry or deformability cytometry [49], to treatment prognosis of sickle cell disease [50].

3. Nuclear to Cytoplasmic Ratio, N/C.

Also termed nucleus-to-cytoplasm ratio, N/C is simply the ratio of the cross-sectional area of the nucleus divided by that of cytoplasm. The N/C is already widely used as a sensitive diagnostic parameter for malignancy, including the staging of cancer [51]. This arises from the fact that one of the most prevalent hallmarks of cancerous cells is the enlargement of the nucleus [52] engendered by increased amounts of chromatin present within malignant cells.

### 2.6. Statistics and Error Analyses

To determine the statistical significance of differences found in measured parameters, we carried out statistical and error analyses using analysis of variance (ANOVA) in Origin (OriginLab, Northampton, MA, USA). ANOVA is a well-established parametric method for means-based comparison of data groups. Origin’s ANOVA algorithm provides an attractive benefit in that it minimizes the probability of type-I errors in statistical analysis. In type-I errors, the null hypothesis is wrongly rejected, leading to the wrong conclusion that results are statistically significant. We performed error analysis using the standard error of the mean (SEM). We carried out at least three independent repeats of every experiment (*N* = 1, *N* = 2, *N* = 3) and report both independent repeats and the averages of the triplicate experiments, as needed, in the main text and in the supplementary information.

## 3. Results

### 3.1. Generated 3D Tissue Spheroids Confirm Microgravity in RCCS

**Figure 2:**
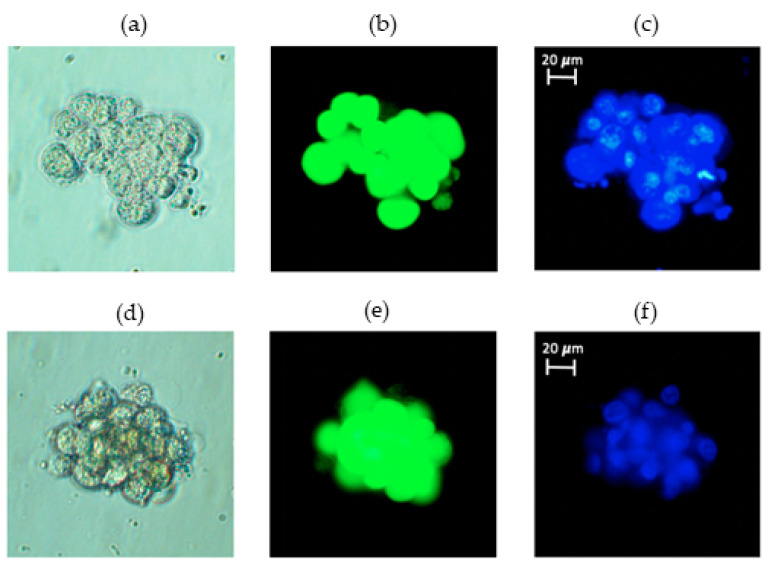
Functional confirmation of RCCS simulation of microgravity via 3D-tissue generation. T98G cells subjected to 72 h of microgravity form 3D spheroids as expected: (left to right) (**a**) phase contrast, (**b**) Calcein stained and (**c**) Hoechst-stained images of the same 3D spheroid consisting of about 20 cells in top panel. The bottom panel has a different spheroid of about 18 cells with similar image characteristics shown: (**d**) phase contrast, (**e**) Calcein-stained and (**f**) Hoechst-stained.

To confirm that our RCCS does produce simulated microgravity, we trypsinized and suspended T98G cancer cells at a concentration of 2 × 10^5^ cells/mL in 10 mL HARVs and rotated these at 15 rpm as already described in the methods section. Figure 2 shows two different 3D spheroids formed from about 20 cells (top panel) and about 18 cells (bottom panel). In Figure 2, the images shown from left to right are (a) and (d) phase contrast, (b) and (e) Calcein-stained (cytoplasm) and (c) and (f) Hoechst-stained (nucleus) images of the same cells in each row. Once brought into suspension, T98G cells usually sink to the bottom of any vessel and adhere to it in less than 6 h, and such adherent cells do not form 3D spheroids but rather a 2D monolayer as shown in Figure S1. Thus, the formation of 3D spheroids inside our RCCS setup, shown in Figure 2 (and in Figure S2, to illustrate multiple spheroids), confirms that our setup does generate microgravity, since such 3D spheroid formation is a well-established effect of microgravity [9,25].

### 3.2. N/C Ratio of Untreated Cells Remains Unchanged following Microgravity

We carried out morphometric analyses on Calcein- and Hoechst-stained images of K562 subjected to 48 h of microgravity and various pharmacological interventions as previously described in the methods section. Of the three morphometric parameters applied (lacunarity, Λ, circularity, *C*, and the nucleus-to-cytoplasm ratio, N/C), only the N/C ratio revealed significant differences in some of the conditions. The morphometric tests were done on 3 to 5 single cells from at least 10 different fields of view taken at 40×, thereby ensuring that over 40 cells were tested for each condition in each experiment, for satisfactory statistical analysis. The lack of significant differences in lacunarity and circularity between different conditions was consistent with our qualitative finding that the entire cytoplasm of the K562 cells did not change much irrespective of the pharmacological intervention. Moreover, the Hoechst stain alone provided sufficient contrast for the extraction of nuclear-to-cytoplasmic ratio as can be seen in Figure 2 and Figure S3.

**Figure 3:**
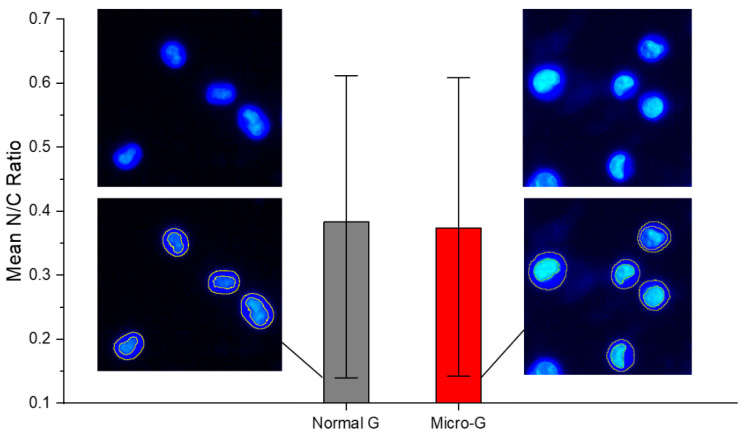
Fluorescence-guided morphometry. Extraction of the nuclear-to-cytoplasmic ratio, N/C from Hoechst-stained images of K562 cells. The Hoechst dye differentially stained the cytoplasm and the nucleus. There was no significant difference in mean N/C ratio between K562 cells in normal gravity (normal G) and those in microgravity (Micro-G). The mean shown here was calculated from morphometry done on one experiment (N1) involving 10 different fields of view and at least 40 cells. This N1 result is representative of all three independent experiments.

As shown in Figure 3, the mean N/C ratio of K562 cells grown in normal gravity (normal G or 1 G) was not significantly different from the N/C ratio of K562 cells subjected to 48 h of simulated microgravity (micro-G or µg). Both conditions show a wide and comparable range of N/C ratios, consistent with the fact that by coupling the DNA content, nuclear size and cell size according to each cell’s stage in the cell cycle, an unsynchronized cell culture maintains a nearly constant but wide range of N/C ratios. This is an interesting result that enables any significant differences obtained in N/C ratios following pharmacological interventions to be attributable to drug response mechanisms. Moreover, cell viability remained above 96% for both 1 G and after 48 h for microgravity (see Table S1).

### 3.3. Hydroxyurea Treated Cells Have a Significantly Altered N/C Ratio in Normal G

**Figure 4:**
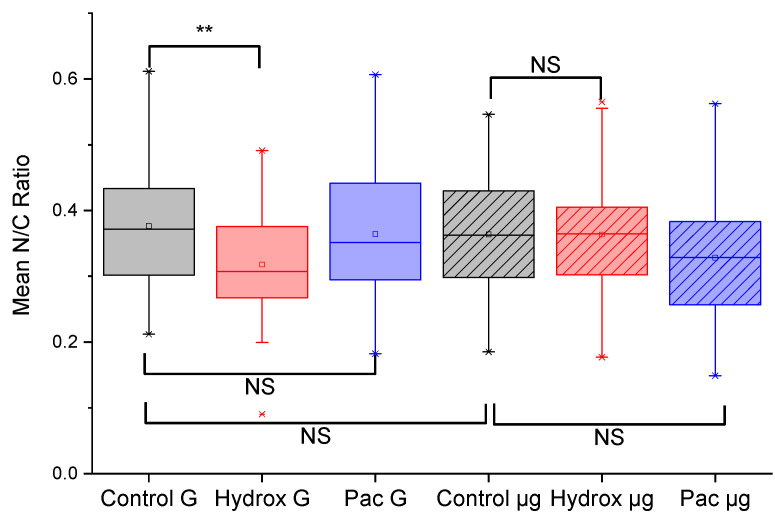
Microgravity-induced changes in cell morphology quantified. The mean nuclear-to-cytoplasmic ratio, N/C, was calculated from morphometry done on three experiments, N1, N2 and N3, each involving 10 different fields of view and at least 40 cells. There is a significant decrease (*p* < 0.01) in the N/C ratio when K562 cells in normal G are treated with hydroxyurea (Hydrox). This significant decrease is lost when K562 cells exposed to 48 h of microgravity are treated with hydroxyurea. In the comparisons, NS means non-significant, ** means *p* < 0.01.

We found a significant decrease (*p* < 0.01) in the N/C ratio between untreated K562 cells in normal G compared to K562 cells treated for 24 h with hydroxyurea also in normal G, as shown in Figure 4. Since hydroxyurea is an antitumor and antileukemic agent that functions by inhibiting mitosis and thereby blocking cell growth [39], the significant decrease in the N/C ratio caused by hydroxyurea treatment can be interpreted as a consequence of a reduction in nuclear size. This interpretation seems plausible since hydroxyurea arrests cells in the S-phase [53], thereby impacting the nuclear size distribution in an unsynchronized cell culture. Arresting cells in the S-phase is so characteristic of hydroxyurea that there is widespread use of it as a cell-cycle synchronizing agent in cell biological experiments [53,54]. Any loss of this response would therefore be a significant indication of a change in this specific drug response.

### 3.4. Microgravity Eliminates the Reduction of the N/C Ratio in Hydroxyurea Treated Cells

To answer the main question raised in this study, namely, whether microgravity might cause alterations in the response of cancer cells to common cancer drugs, we subjected K562 cells to 48 h of simulated microgravity using the RCCS as described in the methods section. As shown in Figure 4, microgravity-subjected K562 cells treated with hydroxyurea (Hydrox µg) had a non-significant change in the N/C ratio compared to untreated microgravity-subjected K562 cells. This was an intriguing result, considering the significant reduction in the N/C ratio in the case of K562 cells in normal G. Moreover, the paclitaxel treated cells showed non-significant changes in the N/C ratio whether in normal G or in microgravity. Since the reduction in the N/C ratio with hydroxyurea treated cells in normal G was rather easy to explain based on the well-known drug mechanism, we further explored evidence for the action of paclitaxel.

### 3.5. Paclitaxel Treated Cells Show No Alterations in the N/C Ratio in Normal G and in Microgravity

**Figure 5:**
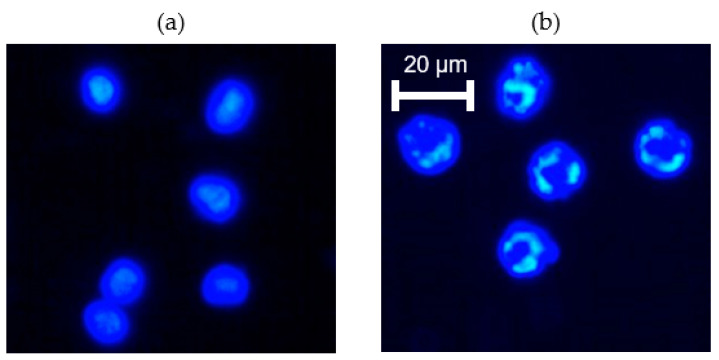
Effects of paclitaxel on cell nucleus revealed via fluorescence imaging using Hoechst dye. (**a**) K562 cells before paclitaxel treatment showing intact nuclei; (**b**) K562 cells 24 h after paclitaxel treatment showing fragmented nuclei. Nuclear fragmentation was found both in the normal G set and in the microgravity set of experiments.

In normal G, the paclitaxel treated cells showed no significant change in the N/C ratio compared to untreated cells (Figure 4). Likewise, following 48 h of microgravity, the paclitaxel treated cells also showed no significant change in the N/C ratio compared to untreated cells (Figure 4). Since paclitaxel causes mitotic cell cycle arrest by stabilizing microtubules [40,41], one would expect the classic formation of micronuclei due to mitotic catastrophe in paclitaxel treated cells. That is exactly what we found in our paclitaxel treated K562 cells (24 h post-treatment), as shown in Figure 5. Nuclear fragmentation was found both in the normal G set of experiments as well as in the microgravity set of experiments. Of course, nuclear fragmentation does not mean a reduction in nuclear size but rather several pieces of the nuclear material instead of one nucleus. Taken together, Figure 4 and Figure 5 indicate that microgravity significantly alters the K562 response to hydroxyurea, but not the response to paclitaxel. Some of the implications of this finding and the need for further work are discussed next.

## 4. Discussion and Conclusions

We have quantified simulated microgravity-induced changes to drug response in K562 cancer cells using fluorescence microscopy and morphometry. Morphometric parameters used included lacunarity, circularity and the nuclear to cytoplasmic ratio, N/C. The N/C ratio was the parameter that showed the most significant changes between the experimental conditions. Our findings have broad implications for reported work in the literature and ongoing research while giving impetus to new research directions. First, the generated 3D tissue spheroids of the T98G cells confirm microgravity in RCCS at our selected 15 RPM, since 3D spheroid formation is a well-ascertained effect of microgravity on adherent cells that have been brought into the suspended state under microgravity conditions [9,25]. Second, the N/C ratio of untreated cells remains unchanged following microgravity. Although this result enabled us to attribute significant differences obtained in the N/C ratios following pharmacological interventions to drug response mechanisms in the cells, it will still be important to reconcile this “non-change” with the microgravity-induced genomic and proteomic differences reported in the literature [55,56]. Third, hydroxyurea treated cells have a significantly (*p* < 0.01) reduced N/C ratio in normal G. Microgravity apparently induces a loss of this reduction. This finding of ours supports the increasing attempts to use simulated microgravity as a tool for drug discovery and development [9,10]. Our fourth and fifth findings that microgravity eliminates the significant reduction of the N/C ratio in hydroxyurea treated cells while the paclitaxel treated cells show no such alterations in the N/C ratio in both normal G and in microgravity, extend recent findings that microgravity alters the effects of chemotherapeutic drugs on cancer cell migration [11,12] in a drug-dependent manner. Based on our current data, we make the educated guess that the early nuclear fragmentation caused by paclitaxel at 24 h, both in 1 G and in µg, leads to non-significant changes in the N/C ratio. However, with hydroxyurea in 1G, there was a significant reduction in the N/C ratio for cells treated with hydroxyurea, compared to untreated. However, in microgravity-subjected K562 cells treated with hydroxyurea, there was a non-significant change in the N/C ratio compared to untreated microgravity-subjected K562 cells, and we see this as an instance of microgravity-induced alteration of drug response, the reasons for which are unclear. Thus, a lot more work needs to be done on various cell types using many different drugs before we can have reliable speculations on the molecular mechanisms by which microgravity induces changes to drug responses in cells. Furthermore, the effects of rotation-induced shear stress will have to be decoupled from the effects of microgravity, via the introduction of a rotational control in the experimental design as done by Mylabathula et al. [38]. Interestingly, the work by Mylabathula et al. [38] also sets the stage for investigating the balance in the cancer-immune axis in addition to drug response changes during exposure to simulated microgravity.

Overall, our results indicate a clear but tentative answer to the overarching question addressed in this work, namely, whether microgravity induces alterations to drug response in cancer cells. The clear and tentative answer is a caveated affirmative, that microgravity induces changes to drug response in cancer cells in a drug-dependent manner. Of course, other cancer cell types need to be tested similarly and many other drugs need to be used. Furthermore, detailed molecular mechanisms will emerge after more parameters are interrogated beyond morphometric characteristics. Our work has set the stage for a varied and variegated exploration of drug response in microgravity conditions, in light of personalized space medicine and earth-based biomedical applications.

## Data Availability

Data is contained within the article and within supplementary material.

## Supplementary Materials

The following supporting information can be downloaded at: https://www.mdpi.com/article/10.3390/life13081683/s1, Figure S1: Images of adherent T98G cells; Figure S2: Microgravity-induced spheroid formation in T98G cells; Figure S3: Steps in morphometry of T98G cells using ImageJ. Table S1: Viability of static control cells in 1G and cells following 48 h of microgravity.
