# Supplementary material for: Simulated Microgravity-Induced Changes to Drug Response in Cancer Cells Quantified Using Fluorescence Morphometry
Source: Life (Basel). 2023 Aug 4;13(8):1683. doi: 10.3390/life13081683 (PMC10455503; doi:10.3390/life13081683)
Supplement: Supplementary file 1 [file life-13-01683-s001.zip › life-2487705-supplementary.pdf]

SUPPLEMENTARY INFORMATION

Microgravity-Induced Changes to Drug Response in Cancer Cells Quantified Using Fluorescence Morphometry

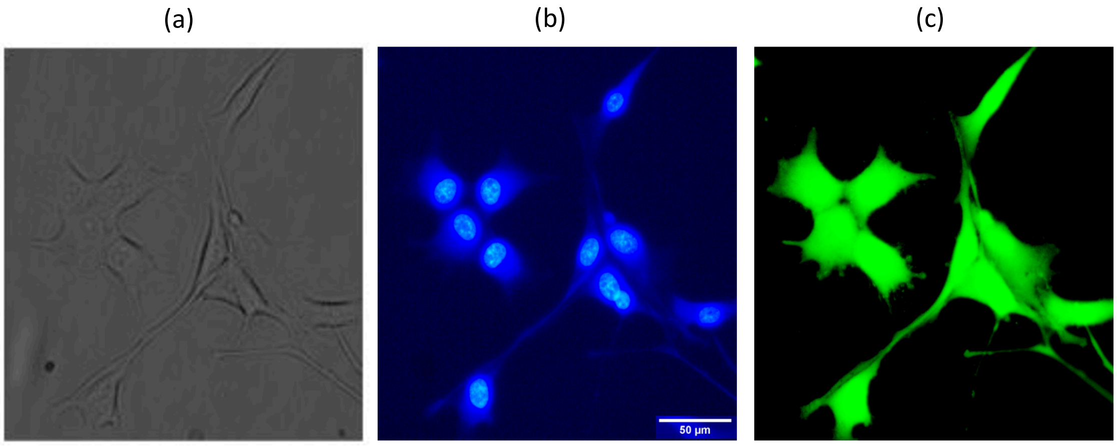

Figure S1. Images of adherent T98G cells. Images of T98G cells at 40x magnification. (a) Phase-contrast image of cells. (b) Fluorescence image of the same cells in (a) under a blue filter, highlighting Hoechst fluorescence. (c) Fluorescence image of the same cells in (a) under a green filter, highlighting Calcein fluorescence.

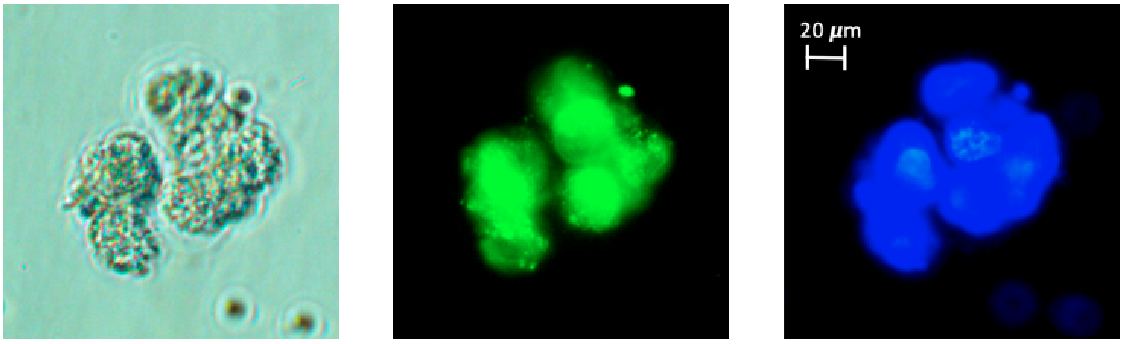

Figure S2. Microgravity-induced spheroid formation in T98G cells. More images of spheroids formed following 72 hours in microgravity.

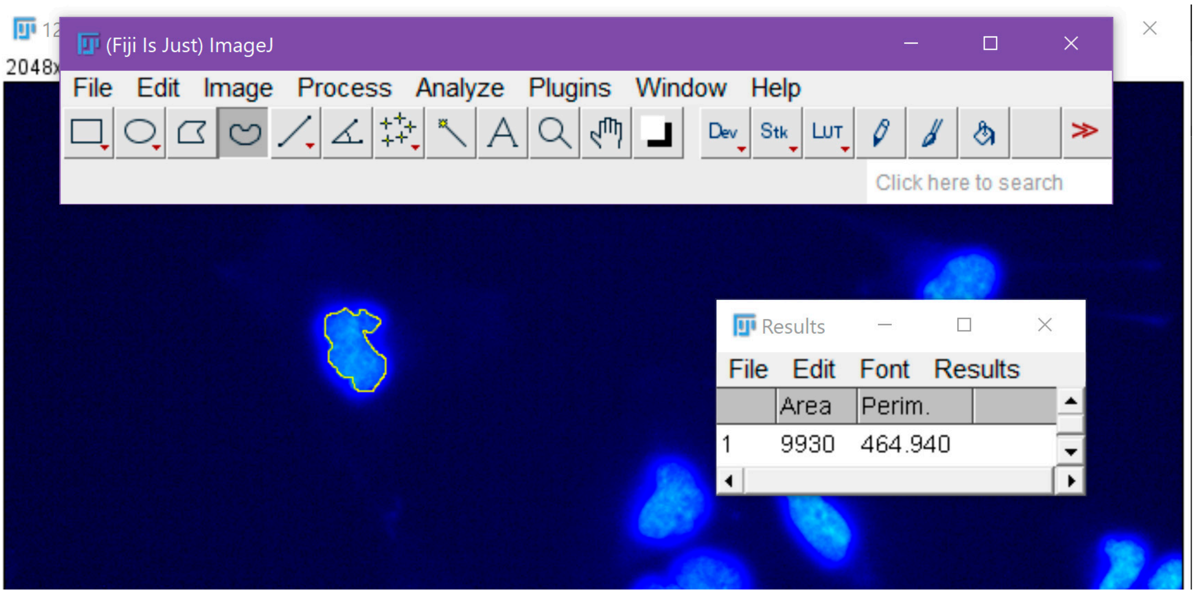

Figure S3. Steps in Morphometry of T98G cells using ImageJ. Hoechst fluorescence image of T98G cells and mapping of regions of interest (here, nucleus). Morphometric data from ImageJ are exported to a spreadsheet for further analysis using OriginLab.

Experiment	1G (after 48 hrs in incubator)	**μ g(after 48 hrs in microgravity)**
**N1**	99.0 ∓1.0%	99.3 ∓1.0%
**N2**	98.5 ∓1.5%	98.0 ∓1.5%
**N3**	96.0 ∓1.5%	96.5 ∓1.5%

Table S1. Viability of static control cells in 1G and cells following 48 hours of microgravity. Both conditions consistently had viability above 96%, optimal for the pharmacological experiments.
